# Empathy in nurse-patient interaction: a conversation analysis

**DOI:** 10.1186/s12912-021-00535-0

**Published:** 2021-01-12

**Authors:** Yijin Wu

**Affiliations:** School of Translation Studies/Editorial Office for Medical Humanities in the Developing World, Qufu Normal University, No.80, Yantaibei Road, Donggang District, Rizhao, China

**Keywords:** Empathy, Nurse-patient interaction, Conversation analysis

## Abstract

**Background:**

Considerable attention has been drawn to empathy in nursing and the concept of empathy has firmly been embedded in nursing discourse. However, little has been known about the details of how nurses express empathy to their patients. In this study, we aim to conduct a qualitative study of actual nurse-patient conversations through which empathy was achieved.

**Methods:**

The data in this study was based on audio-recording of sessions of conversations between participating nurses and patients in two Chinese hospitals. The participants in this study involved 6 female nurses and 14 patients. Based on Bachelor’s (1988) categorization of empathy, this study described and analyzed the actual empathic sequences in nursing conversations in an attempt to demonstrate how nursing empathy was interactionally achieved using the method of conversation analysis. Conversation analysis (CA), focusing on the study of talk in interaction, is a useful method for the qualitative analysis of empathic talk in nursing.

**Results:**

By drawing on prior theoretical work as well as on empathic sequence in nursing, this study described and analyzed some of the conversational resources nurses and patients used in achieving empathy. It has been shown that empathy can be interactionally and sequentially achieved in actual sequences of talk. Specifically, nursing empathy is a collaboratively constructed action instead of the nurse’s own committed action, which is produced in specific interactional contexts.

**Conclusion:**

Conversation analysis is a very useful method for describing and analyzing the nurse-patient interaction, especially for studying empathy in nursing care. The sequences in this study present example of exemplary empathic interaction between nurses and patients, which might shed some light on how nurses express empathy to their patients. Also, this study could help to increase the understanding of the mirco-process of empathy in nursing and contribute to improving nursing communicative skills.

## Background

As a fundamental element for nursing care [1], empathy is closely connected with the caring process in nursing [2]. Nursing empathy could be characterized by nurses’ ability to understand the feeling, experiences or psychosocial ability of their patients [3]. Empathy has an important role to play in establishing a positive nurse-patient relationship and offering favourable nursing care [4]. Nurses who show understanding of their patients’ illness experiences will find their relationship enhanced [5]. Research on nurse-patient communication demonstrates that nurse empathy could provide high-quality care to their patients [6]. Empathy is the essence of all nurse-patient interaction [7–9], and should be seen as an important clinical indicator for offering high-quality nursing care [10].

Moreover, an empathic relationship between nurses and patients could contribute to improved clinical outcomes. Norman (1996) reported that empathy is beneficial to the delivery of care for the elderly mentally ill [11]. Reynolds and Scott (2000) reported that nursing empathy could contribute to patient’s positive responses such as relief from pain, improved pulse, and emotional self-disclosure [12]. Williams (1979) found that the elderly patients received nursing empathy would experience a statistically significant improvement of self-concept [13]. It has also been found that nurse empathy could reduce the cancer patient’s anxiety, depression, and hostility significantly [14].

Considerable attention has been drawn to empathy in nursing and the concept of empathy has firmly been embedded in nursing discourse [15]. However, there have little research available that examines how nursing empathy occur in Chinese nursing care. To the best of our knowledge, this study is the first qualitative research on empathy in nurse-patient communication in Chinese nursing care. So far, Bachelor’s (1988) work on empathy has been considered as one of the most systematic and influential contributions to the study of the types of empathy in psychology [16, 17]. In an analysis of how clients perceive the therapist empathy, Bachelor (1988) identified that therapist empathy can be classified into four types, namely cognitive, affective, sharing, and nurturant empathy [18]. In present study, on the basis of Bachelor’s categorisation of empathy, we will examine if this categorisation is available for identifying and analyzing empathy in nurse-patient communication. It was relatively unproblematic to apply Bachelor’s categorization of empathy to study empathy in other fields, because her system is based on a large scale empirical research [16]. In the present study, using naturally occurring nurse-patient conversation, the author will describe and analyze the interactional sequences whereby empathy is achieved and explore the details of how nurses show empathy to their patients following the framework for categorization of empathy proposed by Bachelor (1988). The research questions were the following: 1). How do nurses and their patients collaborate in producing conversations which could show nursing empathy? 2). How nursing empathy was interactionally achieved in actual sequences of talk?

## Method

### Patient and public involvement

This study was conducted as part of a larger prospective study that deals with the process of nurse-patient communication in China. The data in this study was based on audio-recording of sessions of conversation between participating nurses and patients in two Chinese hospitals. All data in this study consisted of 230 min of nurse-patient conversation, which involved 6 nurses and 14 patients. All nurses involved in this study were female. They aged between 22 to 35 years old. Among the 14 patients, 10 are male, and the remaining 4 patients are female. They aged between 38 to 65 years old. Nurses involved have more than two years of experience in nursing care. The inclusion criteria for patients were 1) ≥18 years of age; 2) clear consciousness; 3) with good communication ability; 3) hospitalized. Informed consent was obtained from all participating nurses and patients. The length of nursing care ranged from 12 to 30 min. The audio-recordings were transcribed verbatim. The spoken data are Chinese, which is translated into English. Here, the data were presented in English.

### Data analysis

Conversation analysis is an approach to the study of the social organization of ‘naturally occurring conversation’, or ‘talk-in-interaction’ through a detailed characterization and analysis of target data collected by audio or video recording equipment [19, 20]. Special focus is given to the identification and description of various types of interactional sequences in the course of social interaction. Sequentiality is characterized by relations of nextness between utterances (turns-at-talk), that is, the speaker’s ongoing utterance displays his or her understanding of the preceding speaker’s utterance [19]. This kind of relation between turns is “endemic to the organization of conversation” [20] and as such, it is the backbone of the possibility of intersubjective understanding between human beings [21, 22]. Conversation Analysis, as the study of talk-in-interaction, should therefore have much to say about empathic interactional sequences in nursing care. Jones (2003) argues that the most notable strength of CA in nursing research lies in its ability to uncover the dynamic interactional order that occurs in much nurse-patient interaction whilst providing some guarantee that ‘analytic considerations will not arise as artifacts of intuitive idiosyncrasy, selective attention or recollection, or experimental design’ [23]. Also, using the CA approach to study nurse-patient interaction offers us a good opportunity to recognize the importance of managing communication exchanges in nursing care such as admission interviews with the aim of successfully integrating the patient’s experience into the nursing assessment [24]. Methodologically speaking, qualitative approach to empathy will be able to build a sound basis for theory development in this area of nursing care [25].

In this study, the detailed transcripts involve the sequential nature of nurses’ empathic response to clients’ utterances. The data were transcribed using Jefferson’s system [26]. Selecting target instances is also an important step in conducting conversation analytical study [27]. Specifically, each extract should contain utterance showing empathy and its preceding and following turns related to empathy.

Using the method of conversation analysis, this study would conduct a turn-by-turn analysis of how these interactional practices display empathy in sequences. Data analysis involves not only what is occurring in the extract but also how it is relevant to the overall interaction. Particular actions which occurred prior to the selected excerpt are also discussed. Describing particular actions which occurred prior to the selected excerpt could contribute to a better understanding of why and how nurses express empathy.

## Results

### Cognitive empathy

Utterances used by the therapist to demonstrate understanding of the thoughts, feeling, or behaviour of the patient are identified as “cognitive empathy” [18]. An instance of cognitive empathy was shown in extract 1 where the nurse offers a candidate understanding of what the patient is feeling. Extract 1 is taken from an interaction between a female nurse and a male patient who suffered from pneumoconiosis. This session occurs when the nurse makes her rounds. In this sequence, it is the nurse that initiates an exchange by making an assumption that the patient seems not to be happy. The nurse has arrived at this assumption through the observation that the patient has a negative facial expression. In line 2, the patient makes an explanation why he does not look happy. In the ensuing talk, the nurse responds to the patient’s troubles talk with an acknowledgment token“En”*(uhm*) repeated twice, displaying his supportive orientation toward the troubles talk in line 2. Immediately following this, the nurse’s demonstration of cognitive empathy occurs in line 4, where the nurse offers an alternative way to pin down the patient’s ongoing feeling, that is, replacing ‘cannot feel happy’ with ‘feel down’. In this sense, the nurse attempt to provide a more specific description of the patient’s unhappiness following the onset of disease and thus seems to have more accurate knowledge about the contents of the patient’s ongoing feeling. In line 5, the nurse encourages the patient to keep a good mood which could contribute to his recovery. Using an agreement token “right”, the patient shows his acceptance of empathy and encouragement the nurse offers in lines 4 and 5. In this extract, it seems that the nurse accurately perceives and understands the patient’s feeling and communicates that understanding back to the patient successfully, thus displaying cognitive empathy.

#### Extract 1

01 Nurse: You seems not to be happy.

02 Patient:My health got (.) really bad, I cannot feel happy.

03 Nurse: Uhm uhm.

04 people feel down when they are (.) not in a good health,

05 but you should keep in a good mood that can help aid your recovery.

06 nurse: right

Extract 2 shows a sequence where the nurse displays a candidate understanding of what the patient is thinking. In line 1, the nurse suggests that the patient should not do that work again. In line 2, the patient responds with a full acknowledgment of the nurse’s recommendation, which can be shown by the fact that once he goes back home, he will change his job to another one. In what follows, the nurse gives the patient a supportive feedback on what he has said. In lines 4 and 5, the patient says that health is much more important than money and thus he will change his job after his hospital discharge, implying that the way for him to earn money was at the expense of his health. The nurse’s expression of cognitive empathy occurs in line 6, where she provides an upshot formulation of the patient’s preceding utterance. Several CA studies have suggested that formulation is one conversational action that regularly functions as a vehicle for empathetic responses [27–29]. In a formulation, one speaker (in this case, the nurse) shows his or her understanding of the other’s (in this case, the patient’s) preceding utterances by proposing a rephrased version of it [30]. In this study, formulation refers to the act that nurses try to show a candidate understanding of what has just been said by clients. By means of formulation, the nurse rephrases the patient’s current thought that health is much more important than money. In line 7, the patient receives and acknowledges the patient’s expression of empathy by means of laughing. What the patient says in line 8 indicates that he shows high degree of agreement with the empathic response the nurse made in line 6.

#### Extract 2

01 Nurse: Don’t do that work again.

02 Patient: Uh, once I go back home, I will (.) change my job.

03 Nurse: Change it to another one, change it to another one.

04 Patient: Uh, health is much more important than money.

05 and I will change-change my job after my hospital discharge.

06 Nurse: Health is a priceless wealth.

07 Patient: Ha ha ha.

(0.7)

08 It does not make any sense to earn much more money without health

#### Sharing empathy

Sharing empathy is understood as the sequences where the therapist displays that he/she has something in common with the patient, specifically, his/her personal opinions or experiences are similar to the patient’s ongoing situation and thereby the patient does not feel alone [18]. In Extract 3, a patient who has trouble in sleeping is discussing his symptom with the nurse. The nurse initiates the topic by an indirect speech act, that is, she has heard that the patient had trouble in sleeping. The information about the patient’s sleep problem may come from the attending doctor or the medical record. In line 2, the patient offers a candidate understanding of the cause for his symptom. In line 3, the nurse first repeats the utterance produced by the patient in line 2. Here, it is an extreme case formulation “quite right”, justifying the patient’s self-assessment of the cause for his sleeping problem. Immediately following the justification, the nurse shows that she also has the same problem as the patient has, that is, she also has trouble in sleeping in that she works too much at night, thereby expressing ‘sharing empathy’. In this sense, by expressing a sense of shared understanding, the nurse may prevent the patient from feeling alone and isolated. Towards the end of line 4, the nurse chooses to use “We”, which is a “we-inclusive” pronoun, including both the nurse and the patient, which aims to shorten the psychological distance between them and bind them into a temporary alliance, contributing further to indicating her shared understanding of the patient’s problem. At this point, it can be seen that the nurse and patient are in perfect alignment with each other in terms of sleeping problem. It could be suggested that sharing empathy can strengthen the nurse-patient relationship and put them on equal footing.

#### Extract 3

01Nurse: I heard that you have not been (.)sleeping well, right?

02Patient: Uhm, (0.5) it may be connected with (.) working at night.

03 Nurse:Quite right, because of working at night too much, I also have sleeping problem.

04 We are prone to sleep problems due to working too much at night.

5 Patient: Right, right.

#### Affective empathy

Affective empathy occurs in the sequences where the therapist shows that he/she partakes of the same feelings the client is personally experiencing at that moment [18]. In the following extract, an instance of affective empathy is presented. In this instance, the patient has been hospitalized for almost one month. In line 1, the patient asks the nurse when he could be discharged from the hospital. In the same turn, he continues to say that he cannot eat well and sleep well, which indicates that he is annoyed over hospitalization. Here, the patient’s negative emotion hides behind the literal meaning of his utterance. Coulehan et al’s (2001) report that in some cases the patient present an indirect expression of suffering or emotion,which are embedded in quasi-factual statements [31]. In this sequence, the nurse does not ignore the patient’s underlying concerns, but empathetically responds to his negative emotion. In lines 3–5, the nurse says that she experienced a similar troubling situation as the patient last year, namely, being hospitalized for three weeks. Thus, she could deeply understand that it is inconvenient for the patient to be in the hospital for a long time. It is clear that the nurse share the same feeling as the patient, namely, being uncomfortable in the hospital for a long time. In this way, the patient seems to describe it as ‘your pain in my heart’. This could contribute to help the nurse build emotional connections with the patient. The nurse thus expresses her empathy and partakes in the patient’s feelings. The patient’s “ye:ah” in line 6 serves to indicate that the patient has received the nurse’s expression of empathy and also functions as a confirmation of the nurse’s statement that she also feels inconvenient. After this, the patient continues to say an utterance “all things are not convenient”, showing that what the patient feels is consistent with the empathic statement the nurse made in line 3. It thus can be seen that communicating empathy is just as important as care delivery.

#### Extract 4

01 Patient: When could I (.) be discharged from hospital?

02 I cannot eat well and sleep well in the hospital.

03 Nurse:I were hospitalized for three weeks last year.

04 I could (.) deeply understand that it is downright inconvenient for you to be in.

05 the hospital for a long time.

06 Patient:ye::ah, all things are not convenient

### Nurturant empathy

The final type of empathy that will be presented and described here is that of ‘nurturant empathy’, which is characterized by the therapist being supportive, security-providing or totally attentive [15]. In the following extract, an instance of nurturant empathy is demonstrated. In line 1, the patient tells the nurse that she worries about the surgery that will be performed tomorrow. The nurse follows up on this by stating that she feels assured because the doctor, Prof. Zhang, who will do surgery for her tomorrow, is a skillful practitioner. It appears that the nurse’s contribution conceives the patient’s concern as unreasonable and thus the nurse is confident about providing security to tomorrow’s surgery. In line 4, the patient uses two minimal responses “uh” and an acknowledgment token “that is right” to show her receipt of the nurse’s expression of nurturant empathy. In line 5, the nurse says that 300 patients similar to her condition have received successful surgeries in this department last year. The nurse attempts to convince the patient that she would just like the other 300 cured patients in performing her surgery. In this sense, drawing on the previous successful experiences of other patients, the nurse is very confident about the patient’s surgery. In other words, what the nurse states here contributes to displaying her optimistic stance toward tomorrow’s surgery, which in itself may achieve a therapeutic effect [32]. The patient’s utterance “If so, I will not be worried about tomorrow’s surgery.” in line 7 can be seen as a favorable response to the nurse’s empathic utterance in lines 5 and 6, and as an indication of nurturant empathy as a good nursing strategy achieving good effect on the patient’s concern.

#### Extract 5

01Patient: I am going to (.) receive a surgery tomorrow. I am very scared.

02Nurse: Please be at ease, Prof. Zhang will perform the surgery (.) for you.

03 He is a skillful doctor

04Patient: Uhm uhm, that’s right.

05Nurse: 300 patients similar to your condition are at our department last year.

06 They all have received successful surgery

07Patient: Uh, (0.5) if so, I will not be worried about tomorrow’s surgery.

## Discussion

Empathy is regarded as an important component in nursing care. However, little work has been done to examine how nurse and patient interact with each other in sequences of talk through which empathy is achieved. As Jones (2003) puts it, CA is a research approach that could accurately capture the contribution of both participants within nurse-patient interaction. CA could reveal the interactional process of empathy in interaction [30]. Thus, the method of conversation analysis is a useful analytical approach to the study of empathy in nursing. In this study, drawing on the method of conversation analysis, four types of empathic interactional sequences are characterized and analyzed, namely cognitive empathy, affective empathy, sharing empathy and nurturant empathy. Our study suggests that “empathy” establishes a caring environment in which nurses not only express understanding of what the patient is experiencing, but also aligns with patients. The present study suggests that to a certain degree empathy could contribute to a smooth sequential development and improved nursing outcome. The sequences in this study present example of exemplary empathic interaction between nurse and patient and the naturally occurring data provides very useful guidelines for professional development of clinical nurses. It is no doubt that CA provides a new way for observing and understanding nurse-patient interaction. It unfolds the sequential organization in the nurse-patient in detail and explicates the practices and interactional sequences through which the nursing care is carried out. It has also been shown in our study that empathy in nursing care can be interactively achieved in actual sequences of talk which was produced by the nurse’s and patient’s collaborative teamwork. A conversation analytic approach presents a turn-by-turn analysis of how empathy unfolds in the course of nurse-patient interaction.

### Limitations

The method of conversation analysis used in this study has several limitations. For instance, we are not able to relate the use of conversational resources to the interactional outcomes, i.e. in terms of patient’s satisfaction. Furthermore, the research findings in this study do not concern all aspects of empathy in nursing. This study just addresses aspects of empathy that characterized by Bachelor (1988) [18]. In other words, this study does not examine other types of empathy which are not covered by Bachelor’ (1988) categorization. Moreover, there were only female nurses involved in this study, which may not have demonstrated the male nurses’ way of expressing empathy. Another limitation concerns data collection which was only conducted in two hospitals, thus raising concerns of displaying nurses’ way of expressing empathy working in different levels of hospitals.

## Conclusion

CA is an inductive, micro-analytic, and predominantly qualitative method for studying language as it is used in social interaction. The microanalytic approach used in this study is available for understanding the interactional features of nursing empathy. Specifically, we could have a clear understanding of how nurse empathy was initiated, how nurse empathy was expressed and how nurse empathy was responded to by patients’ talk. In this sense, Conversation analysis is a very useful method for describing and analyzing the nurse-patient interaction, especially for studying empathy in nursing care. Thus, we call for more attention to be paid to the role of conversation analysis in nursing care.

## Data Availability

The transcribed data are available on reasonable request from the corresponding author.

## Abbreviation

CA: Conversation analysis
